# Night shift work and the acceleration of chronic kidney disease onset: dose–response relationships, interactions with cardiometabolic genetic risk, and metabolomic mediators

**DOI:** 10.5271/sjweh.4296

**Published:** 2026-07-01

**Authors:** Xianglian Cai, Yiwei Zhang, Ziliang Ye, Yanjun Zhang, Sisi Yang, Xiaoqin Gan, Hao Xiang, Yu Huang, Yiting Wu, Dan Chen, Xiaolong Liang, Xianhui Qin, Yuanyuan Zhang

**Affiliations:** 1 Division of Nephrology, Nanfang Hospital, Southern Medical University, Guangzhou, China.; 2 National Clinical Research Center for Kidney and Urological Disease, Nanfang Hospital, Guangzhou, China.; 3 State Key Laboratory of Multi-Organ Injury Prevention and Treatment, Guangzhou, China.; 4 Guangdong Provincial Institute of Nephrology, Guangzhou, China.; 5 Guangdong Provincial Key Laboratory of Renal Failure Research, Guangzhou, China.

**Keywords:** cardiometabolic disease, metabolomics, polygenic risk score, shift worker

## Abstract

**Objectives:**

The association between night shift work and chronic kidney disease (CKD) risk remains uncertain. We investigated the associations of current/lifetime night shift work with incident CKD, assessed joint effects with genetic susceptibility, and explored the mediating roles of obesity and metabolomic alterations.

**Methods:**

Utilizing UK Biobank data, current (N=242 721) and lifetime (N=63 659) night shift duration/frequency were assessed via questionnaires. The primary outcome was incident CKD. Polygenic risk scores (PRS) for cardiometabolic diseases (diabetes, hypertension, and cardiovascular disease) were calculated. Plasma metabolomics (249 measures, N=122 681) were analyzed.

**Results:**

Over 13.7 years of follow-up, 5654 incident CKD cases were identified. Compared to day workers, those who usually or always worked night shifts had a 25% higher CKD risk [adjusted hazard ratio (HR_adj_) 1.25, 95% confidence interval (CI) 1.10–1.41] and developed CKD 2.06 years earlier (95% CI 0.75–3.25). Lifetime night shift exposure showed consistent dose–response relationships, with 16–17% increased risks for either ≥5 cumulative years (adjusted HR 1.17, 95% CI1.01–1.36) or ≥3 monthly night shifts (adjusted HR 1.16, 95% CI1.01–1.33). Participants with both usual/always night shift exposure and high PRS for cardiometabolic diseases exhibited the greatest CKD risk. Obesity-related parameters (body mass index and waist circumference) mediated 14.7–14.8% of the observed night shift–CKD association. A novel 9-metabolite signature reflective of night shift mediated 5.47% of this association, primarily through disrupted fatty acid metabolism.

**Conclusions:**

Night shift work exhibits a dose-dependent association with CKD risk, exacerbated by cardiometabolic genetic predisposition and partially mediated through metabolic dysregulation and obesity. These findings underscore the need for workplace interventions targeting shift scheduling and metabolic health among high-risk workers.

Chronic kidney disease (CKD) is a leading cause of global mortality, with an age-standardized death rate of 12.2 per 100 000 population (1). Identifying novel modifiable risk factors is therefore a public health priority. Night shift work, an increasingly prevalent feature of modern employment, has been associated with cardiovascular disease (CVD), diabetes, and hypertension (2–4). While emerging evidence suggests a link between shift work and CKD (5), prior studies have largely treated shift work as a binary exposure, leaving critical gaps in understanding the specific impact of night shift work, particularly its current frequency, lifetime cumulative duration, and long-term patterns, and whether a dose–response relationship exists.

Night shift work may promote kidney injury through sleep deprivation and circadian disruption, which can trigger metabolic dysregulation, neuroendocrine activation, and systemic inflammation – cascades that ultimately contribute to renal impairment. These disturbances may also promote obesity, particularly central adiposity (6, 7), a well-established risk factor for CKD. The recently articulated cardiovascular-kidney-metabolic (CKM) syndrome framework highlights the interconnected pathophysiology of CKD, CVD, and type 2 diabetes (T2DM) (8), raising the possibility that genetic susceptibility to these conditions may interact with night shift exposure to amplify CKD risk. However, this gene–environment interaction hypothesis remains untested in prospective studies.

Metabolomics offers a powerful tool to capture systemic biological perturbations along these pathways. Although it has provided insights into cardiometabolic diseases (9, 10), the metabolomic alterations induced by night shift work and their potential mediating role in CKD development have not been systematically investigated. Identifying such alterations could illuminate intermediate mechanisms and reveal novel biomarkers for early detection of shift work-related kidney injury.

To address these gaps, we used UK Biobank data to: (i) examine the associations of current and lifetime night shift work with incident CKD, including dose–response relationships; (ii) evaluate the independent and joint effects of night shift work and polygenic risk scores (PRS) for CKD, T2DM, hypertension, and CVD; and (iii) characterize night shift-related metabolomic profiles and quantify the mediating roles of obesity and metabolomic alterations in the night shift-CKD risk association. By integrating detailed exposure histories, genomics, and metabolomics, this study aims to provide mechanistic insights and identify high-risk subgroups for targeted prevention.

## Methods

### Study design and participants

The UK Biobank is a prospective cohort study that enrolled over 500 000 participants aged 37–73 years from 22 assessment centers across England, Wales, and Scotland between 2006 and 2010. Detailed study procedures have been published previously (11–14). Ethical approval was obtained from the North West Multicenter Research Ethics Committee (Reference: 11/NW/0382), and all participants provided written informed consent.

From 286 164 participants with complete baseline employment data, we excluded those with missing CKD status information (N=25 929), prevalent CKD at baseline [estimated glomerular filtration rate (eGFR) <60 mL/min/1.73 m^2^ or UACR ≥30 mg/g; N=14 776], or missing genetic risk score for eGFR (N=2738), yielding a final analytical cohort of 242 721 participants. Among these, 63 659 completed the lifetime employment questionnaire and were included in lifetime exposure analyses; their characteristics were similar to the overall cohort (supplementary material, www.sjweh.fi/article/4296, table S1). A subset of 122 681 participants had complete plasma metabolomics data (supplementary figure S1).

### Assessment of night shift work

Night shift exposure was assessed using two complementary approaches: current night shift status at baseline and lifetime cumulative night shift history from the 2015 follow-up questionnaire.

### Current night shift work

Shift work was defined as any schedule extending beyond conventional daytime hours (09:00–17:00 hours), including afternoons, evenings, nights, or rotating shifts. Employed participants (including self-employed individuals) reported their shift work status via the baseline touchscreen questionnaire. Those responding “sometimes”, “usually”, or “always” were further asked whether their work involved night shifts (defined as working through normal sleeping hours, 24:00–06:00 hours). Based on these responses, we categorized participants into three mutually exclusive groups: (i) day workers (never/rarely shift work, reference); (ii) shift workers with rare/some night shifts; and (iii) shift workers with usually/always night shifts (15).

### Lifetime night shift work

At the 2015 follow-up, participants completed the online healthy work questionnaire (16), which collected detailed occupational histories for all paid jobs lasting ≥6 months and ≥2 days (or 15 hours) per week after full-time education. For each job, participants reported employment duration and monthly night shift frequency. From these data, we derived two quantitative exposure metrics up to the questionnaire completion date: cumulative lifetime duration of night shift work (years) and average monthly night shift frequency (shifts/month). Based on prior study (17), we stratified lifetime night shift history into mutually exclusive categories by duration (0 [never night shift work], <5, ≥5 years) and frequency (0 [never night shift work], <3, ≥3 nights/month), with supplementary analyses using median cutoffs (10 years, 7 nights/month).

### Plasma metabolite profiling

Plasma metabolomic profiling was conducted in a randomly selected subset of 275 000 participants using a high-throughput nuclear magnetic resonance (NMR) platform (Nightingale Health Ltd, Helsinki, Finland). This assay quantified 249 metabolic measures, including 168 absolute concentrations and 81 derived ratios, covering amino acids, fatty acids, lipoprotein subclasses, cholesterol subtypes, and inflammatory markers. All metabolite measures were log-transformed to normalize distributions and standardized to z-scores prior to analysis.

### Laboratory measurements

Blood and urine samples were collected and processed following standardized protocols (18). Serum creatinine, urinary albumin, and urinary creatinine were measured at a central laboratory. Estimated glomerular filtration rate (eGFR) was calculated using the CKD-EPI equation (19), and the urine-albumin-to-creatine ratio (UACR) was derived from measured concentrations.

### Covariate assessment

Covariates were selected based on prior literature (20, 21) and directed acyclic graphs (supplementary figure S2). Baseline characteristics were collected via standardized questionnaires, including demographic factors [age, sex, ethnicity, Townsend deprivation index (TDI), education level], anthropometric measures [body mass index (BMI), waist circumference], lifestyle factors (smoking, alcohol consumption, physical activity, sleep patterns), and medical history.

Hypertension was defined as self-reported diagnosis, antihypertensive medication use, or blood pressure ≥140/90 mmHg (22). Diabetes was defined as self-reported diagnosis or HbA1c ≥48 mmol/mol. Hypercholesterolemia was defined as self-reported hyperlipidemia or lipid-lowering medication use (23).

Physical activity was categorized by weekly frequency of moderate-to-vigorous exercise, with optimal activity defined as >4 days/week. Sleep quality was assessed using a composite score (range 0–5) incorporating five healthy sleep behaviors (24); higher scores indicated more favorable sleep patterns. Social isolation was evaluated using a three-point index (range 0–3) based on living situation, social contact frequency, and social activity participation, with scores of 2–3 classified as socially isolated (25).

### Study outcomes

The primary outcome was incident CKD, defined using ICD-10 code N18 (26, 27). Cases were identified through multiple sources, including hospital admission records, death registry data, primary care records, and participant self-reports.

Follow-up duration was calculated from the baseline assessment to the earliest occurrence of CKD diagnosis, death, loss to follow-up, or the end of the follow-up period.

### Polygenic risk score calculation

Genotype quality control and imputation procedures for the UK Biobank have been described previously (28). A weighted PRS for elevated eGFR was computed using 263 genome-wide significant single nucleotide polymorphisms (SNP) associated with eGFR (29, 30). Standardized PRS for T2DM, hypertension, and CVD were obtained from the UK Biobank. For each PRS, participants were categorized into high and low genetic risk groups based on the median value.

### Statistical analysis

*Baseline characteristics.* Baseline characteristics were summarized as mean [standard deviation (SD)] for continuous variables and frequencies (percentages) for categorical variables. Differences across current night shift work status (day workers, shift with rare/some night, shift with usually/always night) were assessed using ANOVA and chi-square tests, as appropriate.

*Longitudinal association analyses.* Cox proportional hazards models were used to estimate hazard ratios (HR) and 95% confidence intervals (CI) for the association between night shift exposure and incident CKD. Exposure variables included current night shift status (day workers [reference], shift with rare/some nights, shift with usually/always nights) and lifetime night shift history, analyzed both continuously and categorically by duration (0, <5, ≥5 years) and frequency (0, <3, ≥3 nights/month). Model 1 was adjusted for age, sex, ethnicity, TDI, and education level. Model 2 further adjusted for BMI, physical activity, smoking status, alcohol consumption, baseline cardiometabolic diseases (hypertension, diabetes, and hypercholesterolemia), eGFR, UACR, and PRS for elevated eGFR.

Using the fully adjusted model (model 2), restricted cubic spline (RCS) models were fitted to visualize potential nonlinear dose–response relationships between lifetime night shift duration/frequency and CKD risk. We further examined the joint effect of lifetime night shift duration and frequency on incident CKD, estimated differences in time to CKD onset (in years) across exposure categories using Laplace regression, and generated adjusted cumulative incidence curves with the “adjustedCurves” R package (31).

*Effect modification and sensitivity analyses.* Stratified analyses and interaction tests were conducted based on prior evidence and biological plausibility. Sensitivity analyses included: (i) excluding CKD cases within the first two years; (ii) complete-case analysis; (iii) omitting adjustment for baseline cardiometabolic diseases; (iv) restricting to participants no longer employed; (v) additional adjustment for job type, sleep score, weekly working hours, and social isolation; and (vi) Fine-Gray competing risk models accounting for mortality.

*Genetic analyses.* RCS models were used to examine dose–response relationships between PRS for cardiometabolic diseases and CKD risk. Joint effects and interactions between genetic risk and current night shift exposure were also evaluated.

*Metabolomic and mediation analyses.* A three-step metabolomic analysis was performed. First, multivariable generalized linear models identified metabolites associated with current night shift exposure (treated as a continuous ordinal variable) at Bonferroni-corrected significance (P<0.05). LASSO regression with 10-fold cross-validation selected the most robust biomarkers to derive a weighted metabolic signature score. Second, Cox models with RCS assessed the dose–response relationship between this signature score and CKD risk. Third, mediation analysis using the “mediation” R package with 1000 bootstrap iterations quantified the proportion of the night shift-CKD risk association mediated by the metabolic signature score and by obesity-related indicators (BMI, waist circumference). All models were adjusted for covariates in model 2.

All statistical analyses were performed using R software (version 4.1.1), with a two-sided P<0.05 considered statistically significant.

## Results

### Baseline characteristics of study participants

Of 242 721 participants (mean age 52.6 years; 48.7% male), 82.8% were day workers, 13.4% had rare/some night shifts, and 3.8% had usually/always night shifts. Compared with day workers, those with more frequent night shifts were younger, more likely male and non-White, and had higher socioeconomic deprivation, adverse cardiometabolic profiles, lower education, greater social isolation, and poorer sleep quality (table 1).

**Table 1:** Baseline characteristics of study participants by current night shift work status.[SD=standard deviation;]

Current night shift work characteristics		Day workers			Shift with rare/some night			Shift with usually/always night	
		N (%)	Mean (SD)		N (%)	Mean (SD)		N (%)	Mean (SD)
Age (years)		52.83 (7.06)			51.92 (6.96)			51.16 (6.79)	
Sex									
	Female	105 959 (5 2.7)			15 015 (46.3)			3442 (37.0)	
	Male	95042 (47.3)			17 391 (53.7)			5872 (63.0)	
White		191 889 (95.5)			29 309 (90.4)			8154 (87.5)	
Townsend deprivation index			-1.53 (2.91)			-0.64 (3.25)			-0.48 (3.27)
Body mass index (kg/m^2^)			27.02 (4.55)			27.86 (4.87)			28.30 (4.79)
Education									
	College or university degree/vocational qualification	10 3027 (51.5)			13 014 (40.6)			3187 (34.6)	
	National examination at age 16	53 909 (27.0)			11 049 (34.4)			3673 (39.9)	
	National examination at age 17–18	25 073 (12.5)			3730 (11.6)			970 (10.5)	
	Others	18 006 (9.0)			4280 (13.3)			1373 (14.9)	
Moderate/vigorous physical activities >4 days/week		104 707 (54.2)			20 112 (65.9)			5885 (68.5)	
Smoking status									
	Current	19 421 (9.7)			4770 (14.8)			1594 (17.2)	
	Never	117 075 (58.4)			17 259 (53.5)			4909 (52.9)	
	Previous	64 030 (31.9)			10 257 (31.8)			2784 (30.0)	
Alcohol consumption (times per week)									
	<1	52 328 (26.0)			10 897 (33.7)			3502 (37.7)	
	1–2	55 447 (27.6)			9033 (27.9)			2771 (29.8)	
	3–4	51 688 (25.7)			6962 (21.5)			1932 (20.8)	
	>4	41 438 (20.6)			5489 (17.0)			1095 (11.8)	
Sleep score			3.12 (0.98)			2.96 (1.02)			2.73 (1.05)
Social isolation		28 915 (14.5)			5438 (17.1)			1664 (18.2)	
History of disease									
	Hypertension	92 518 (46.2)			15 593 (48.4)			4621 (50.0)	
	Diabetes	7110 (3.5)			1467 (4.5)			496 (5.3)	
	High cholesterol	22 198 (11.0)			3919 (12.1)			1160 (12.5)	

### Association between current, lifetime night shift work patterns and incident chronic kidney disease

Over a median follow-up of 13.7 years, 5 654 incident CKD cases (2.3%) were identified. In unadjusted models, compared with day workers, those with usually/always night shifts had the highest CKD risk (HR 1.29, 95% CI 1.14–1.46). In fully adjusted models (model 2), current workers with usually/always night shifts had a 25% higher CKD risk than day workers (HR 1.25, 95% CI, 1.10–1.41). Compared with no lifetime night shift history, risk increased with ≥5 cumulative years (HR 1.17, 95% CI 1.01–1.36) and ≥3 nights/month (HR 1.16, 95% CI 1.01–1.33) (model 2, table 2). Those with both long duration (≥5 years) and high frequency (≥3/month) had the highest risk (HR 1.22, 95% CI 1.04–1.42) (model 2, supplementary table S2). Continuous exposure measures, including per SD and log-transformed night shift variables, showed attenuated but persistently positive associations in model 2 compared to model 1. Further stratification of lifetime exposure using median cutoffs (≥10 years or ≥7 nights/month) yielded consistent results, with the highest risk observed among those in the high-duration/ high-frequency group (model 2, supplementary table S3, figure S4).

**Table 2:** Association between current and lifetime night shift work exposure and incident chronic kidney disease (CKD) risk. [CI=confidence interval; HR=hazard ratio].

		N	Case (%)	Crude model				Model 1 ^a^				Model 2 ^b^		
				HR	95% CI	P for trend		HR	95% CI	P for trend		HR	95%CI	P for trend
Current night shift work														
	Day workers	201 001	4525 (2.3)					Ref				Ref		
	Shift with rare/some night	32 406	859 (2.7)	1.19	1.10–1.28			1.23	1.14–1.32			1.17	1.08–1.26	
	Shift with usually/always night	9314	270 (2.9)	1.29	1.14–1.46	<0.001		1.40	1.24–1.59	<0.001		1.25	1.10–1.41	<0.001
Lifetime night shifts’ duration														
	None	49 831	902 (1.8)					Ref				Ref		
	<5 years	3976	74 (1.9)	1.03	0.81–1.30			1.04	0.82–1.32			0.98	0.78–1.25	
	≥5 years	9852	226 (2.3)	1.28	1.10–1.48	0.001		1.29	1.12–1.50	<0.001		1.17	1.01–1.36	0.054
Lifetime night shifts’ average frequency														
	None	49 831	902 (1.8)					Ref				Ref		
	<3 /month	1683	29 (1.7)	0.95	0.66–1.38			0.90	0.62–1.30			0.84	0.58–1.22	
	≥3/month	12 145	271 (2.2)	1.24	1.08–1.42	0.002		1.27	1.10–1.46	0.001		1.16	1.01–1.33	0.054

ª Adjusted for age and sex, ethnicity, Townsend deprivation index, education. ^b^ Model 1 and additionally adjusted for body mass index, physical activity, smoking, alcohol, baseline diseases (hypertension, diabetes, high cholesterol), estimated glomerular filtration rate, urine albumin-to-creatinine ratio, genetic risk score of estimated glomerular filtration rate.

**Figure 1:**
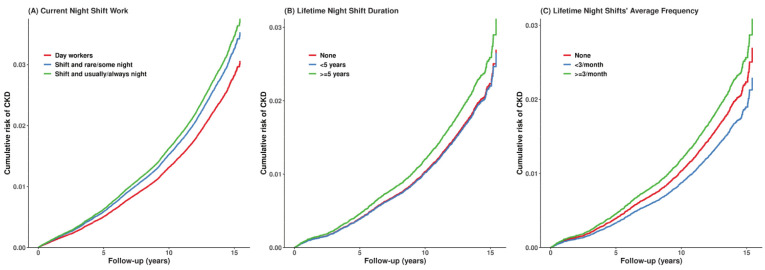
Cumulative incidence of chronic kidney disease (CKD) by night shift work exposure patterns.* (A) Current shift status, (B) lifetime duration, and (C) monthly frequency. *Adjusted for age, sex, ethnicity, Townsend deprivation index, education, body mass index, physical activity, smoking, alcohol, baseline diseases (hypertension, diabetes, high cholesterol), estimated glomerular filtration rate (eGFR), urine albumin-to-creatinine ratio, and genetic risk score of eGFR.

Night shift work also accelerated CKD onset. Compared with day workers, participants with usual/always night shifts developed CKD 2.06 years earlier (95% CI 0.75–3.25). Relative to those without lifetime night shift history, individuals with ≥5 years or ≥3 shifts/month experienced onset 1.24 years (0.18–2.21) and 1.10 years (0.12–2.03) earlier, respectively (supplementary table S4). Cumulative incidence curves further illustrated the graded increase in CKD risk across exposure categories (figure 1).

### Sensitivity and subgroup analyses

The observed associations remained robust across all sensitivity analyses, including exclusion of early CKD cases, complete-case analysis, omission of adjustment for baseline cardiometabolic diseases, restriction to non-employed participants, additional adjustment for occupational and lifestyle factors, and competing risk models accounting for mortality (supplementary tables S5–6).

No significant effect modification was observed by age, sex, BMI, smoking, alcohol, social isolation, chronotype, hypertension, diabetes, hypercholesterolemia, or CKD genetic risk (all P for interaction >0.05) (supplementary figures S5–7).

### High genetic risk to cardiometabolic disorders and night shift work jointly elevate CKD risk

**Figure 2:**
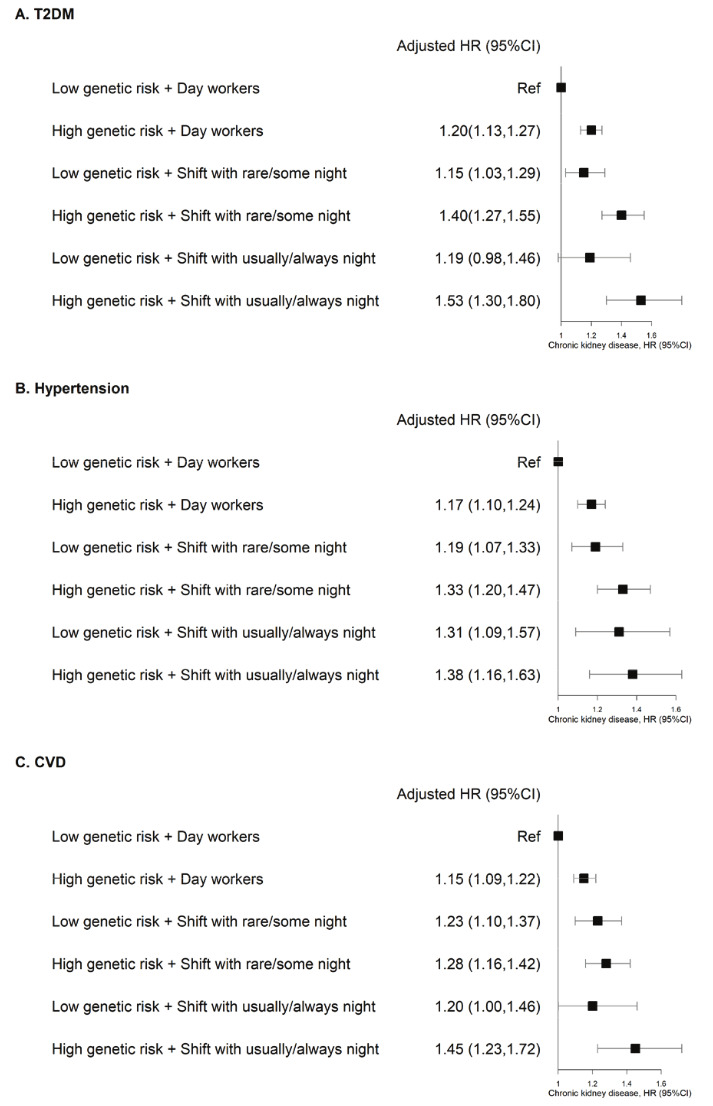
Joint associations of night shift work and genetic susceptibility with chronic kidney disearse (CKD) risk*. Hazard ratios (HR) for incident CKD according to current night shift exposure and polygenic risk for (A) type 2 diabetes mellitus (T2DM), (B) Hypertension, and (C) cardiovascular disease (CVD). *Adjusted, if not stratified, for age and sex, ethnicity, Townsend deprivation index, education, body mass index, physical activity, smoking, alcohol, baseline diseases (hypertension, diabetes, high cholesterol), estimated glomerular filtration rate (eGFR), and urine albumin-to-creatinine ratio. [CI=confidence interval; PRS=polygenic risk score].

Higher PRS for cardiometabolic diseases were independently associated with increased CKD risk. Per SD increment, adjusted HR (HR_adj_) were 1.11, 95% CI 1.08–1.14 for T2DM PRS; HR_adj_ 1.09, 95% CI 1.07–1.12 for hypertension PRS; and HR_adj_ 1.08, 95% CI 1.05–1.11 for CVD PRS (supplementary figure S8). Although no significant statistical interaction was detected between night shift work and genetic risk (supplementary table S7), a clear risk gradient emerged: participants with both frequent night shift exposure and high genetic susceptibility had the highest CKD risk, with HR_adj_ of 1.38–1.53 compared to daytime workers with low genetic risk (figure 2).

### Association of the current night shift-related metabolic signature with incident CKD risk

**Figure 3:**
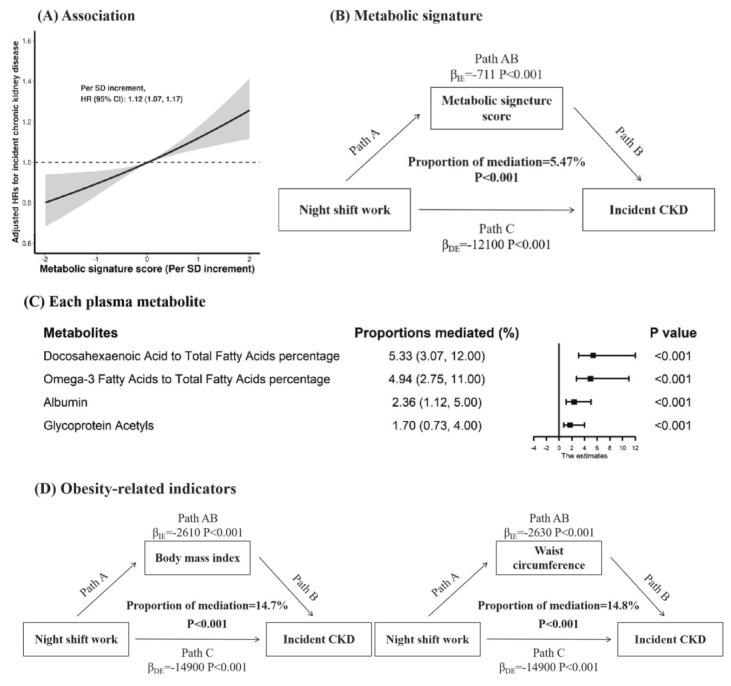
Metabolic signature of night shift work and Its mediating role in chronic kidney disease (CKD) risk.* (A) Dose-response association between the night shift-related metabolic signature score and incident CKD; (B) Proportion of the night shift–CKD risk association mediated by the metabolic signature; (C) Mediating effects of individual metabolites; (D) Mediating effects of obesity-related indicators [body mass index (BMI) and waist circumference]. *Adjusted if not as a mediator, for age and sex, ethnicity, Townsend deprivation index, education, BMI, physical activity, smoking, alcohol, baseline diseases (hypertension, diabetes, high cholesterol), estimated glomerular filtration rate (eGFR), urine albumin-to-creatinine ratio, and genetic risk score of eGFR. [CI=confidence interval; HR=hazard ratio; SD=standard deviation.]

In plasma metabolomic profiling of 122 681 participants, we derived a weighted metabolic signature score comprising nine metabolites that robustly reflected current night shift exposure, including markers of inflammation, lipoprotein metabolism, and fatty acid composition (supplementary table S8). This signature demonstrated a significant dose–response relationship with incident CKD: each SD increment was associated with a 12% higher risk in fully adjusted models (HR_adj_ 1.12, 95% CI 1.07–1.17; figure 3A). Among the nine constituent metabolites, four showed consistent directional associations with both night shift exposure and CKD risk: lower ratios of docosahexaenoic acid to total fatty acids and omega-3 fatty acids to total fatty acids, lower albumin, and higher glycoprotein acetyls (supplementary table S9).

### Mediation analyses of metabolic and obesity-related pathways

In mediation analyses based on fully adjusted models, the metabolic signature score mediated 5.5% (95% CI 2.99–13.00) of the total association between current night shift exposure and CKD risk (figure 3B). Among individual metabolites, mediation proportions were 1.7–5.3%: docosahexaenoic acid ratio (5.3%; 95% CI 3.07–12.00), omega-3 fatty acids ratio (4.9%; 95% CI 2.75–11.00), albumin (2.4%; 95% CI 1.12–5.00), and glycoprotein acetyls (1.7%; 95% CI 0.73–4.00) (figure 3C; supplementary table S9).

Obesity-related indicators showed substantially larger mediation effects. BMI and waist circumference mediated 14.7% and 14.8% of the association, respectively (figure 3D), suggesting adiposity as a more prominent mechanistic pathway than the identified metabolomic alterations.

## Discussion

In this large-scale prospective study, we found that both current and lifetime night shift work were associated with increased CKD risk in a dose-dependent manner and with earlier disease onset. High genetic susceptibility to cardiometabolic diseases compounded this risk, though the adverse effects of night shift work were evident across all genetic backgrounds. Metabolomic profiling identified a nine-metabolite signature of night shift exposure, reflecting alterations in fatty acids, lipoproteins, and inflammatory markers, that partially mediated the observed association. Obesity-related parameters emerged as stronger mediators, accounting for approximately 15% of the total effect.

### Dose–response relationship between night shift work and CKD risk

We observed a dose-dependent association between night shift work and CKD risk, evident for both current and lifetime exposure and consistent across subgroups. These findings extend prior evidence linking shift work to renal dysfunction (5). The accelerated CKD onset – by nearly two years among those with usual/always night shifts – underscores the clinical significance of these exposures. Biological mechanisms may involve circadian disruption and sleep impairment, leading to altered blood pressure rhythms, sympathetic nervous system activation, and metabolic dysregulation (32, 33). The association persisted after adjustment for sleep quality, suggesting effects beyond conventional sleep deprivation, likely involving circadian disruption. Thus, early renal monitoring and interventions should extend beyond sleep improvement alone.

Long-duration (≥5 years) and high-frequency (≥3 shifts/month) night work emerged as high-risk exposures warranting priority intervention. While low exposure showed no significant risk in this study, possibly reflecting subthreshold cumulative damage or compensatory renal adaptation, long-term monitoring remains prudent.

### Genetic susceptibility modifications

Although formal interaction tests were not statistically significant, a clear risk gradient emerged: individuals with both frequent night shift exposure and high polygenic risk for cardiometabolic diseases exhibited the highest CKD incidence. This pattern suggests that night shift work may exacerbate underlying genetic predispositions through circadian disruption and metabolic stress. Importantly, elevated CKD risk was also observed among those with low genetic risk, indicating that low genetic susceptibility does not fully offset the adverse effects of night shifts. These findings support that minimizing night shift work may benefit all workers, irrespective of genetic background.

### Metabolic signature of night shift work and its mediating role in CKD pathogenesis

The metabolomic analysis revealed a nine-metabolite signature of night shift exposure, including fatty acid ratios, albumin, and inflammatory glycoproteins, that provides a biochemical footprint of night shift-related physiological disruption. The inverse associations of omega-3 fatty acid ratios with both night shift exposure and CKD risk are particularly noteworthy, given their known anti-inflammatory and renoprotective properties (34, 35). Mediation analysis suggested that night shift work may impair renal function partly through depletion of protective fatty acids and promotion of a pro-inflammatory state. However, the modest mediation proportion (5.5%) indicates that these metabolomic alterations represent only one component of a complex multifactorial pathway.

### Obesity as a key mediator

Obesity-related parameters emerged as the strongest mediators in our analysis. BMI and waist circumference each accounted for approximately 15% of the night shift–CKD association, nearly three times the proportion mediated by the metabolomic signature. This finding supports the hypothesis that night shift work promotes central adiposity (36), which in turn contributes to renal impairment through hypertension, insulin resistance, glomerular hyperfiltration, and chronic inflammation (37). The prominence of this pathway highlights weight management as a potentially high-yield intervention target for mitigating CKD risk in night shift populations.

### Clinical and public health implications

Our findings carry several implications. First, night shift work history should be considered in CKD risk assessment, particularly for those with high cardiometabolic genetic risk, to inform personalized preventive counseling. Such assessments must be used exclusively for health guidance and must not inform employment decisions or insurance determinations. Second, the identified metabolomic signature may serve as a biomarker for early detection of shift work-related health decline, though further validation is needed. Third, employers and policymakers should prioritize structural interventions: limit long-duration (>5 years) and high-frequency (>3 shifts/month) night work, and provide periodic opportunities for transition to day-oriented roles. Fourth, workplace environments should support healthy behaviors during night shifts, including access to nutritious food, protected break times, and facilities for physical activity. Finally, regular health screenings focusing on renal and metabolic function should be coupled with access to medical resources, workplace accommodations, and professional counseling.

### Strengths and limitations

Key strengths of this study include the large sample size, prospective design, comprehensive assessment of current and lifetime night shift exposure, integration of genetic and metabolomic data, and rigorous adjustment for potential confounders.

Several limitations warrant consideration. First, night shift exposure was self-reported and may be subject to recall bias. Second, despite extensive adjustment, residual confounding from unmeasured factors cannot be ruled out. Third, the long interval between baseline covariate assessment and follow-up lifetime night shift work may lead to temporal misalignment in adjustment. Fourth, the predominantly European-ancestry cohort limits generalizability to other populations. Finally, the observational design precludes definitive causal inference.

### Concluding remarks

This study demonstrates that night shift work is associated with increased CKD risk in a dose-dependent manner, with the highest risk observed among individuals with high cardiometabolic genetic susceptibility. A novel nine-metabolite signature of night shift exposure partially mediated this association, while obesity-related parameters emerged as stronger mediators, accounting for approximately 15% of the total effect. These findings provide new insights into the biological pathways linking night shift work to CKD and highlight potential targets for prevention, including weight management and metabolomic monitoring. Future research should validate these findings in diverse populations and evaluate interventions to mitigate the adverse health effects of shift work.

## Supplementary material

Supplementary materials

## Data Availability

The UK Biobank data are available on application to the UK Biobank, and the analytic methods, and study materials that support the findings of this study will be available from the corresponding authors on request.
