# Supplementary material for: Night shift work and the acceleration of chronic kidney disease onset: dose–response relationships, interactions with cardiometabolic genetic risk, and metabolomic mediators
Source: Scand J Work Environ Health. 2026 Jun 26;52(4):442–51. doi: 10.5271/sjweh.4296 (PMC13349410; doi:10.5271/sjweh.4296)
Supplement: Supplementary materials [file SJWEH-52-442-S001.pdf]

Night shift work and the acceleration of chronic kidney disease onset: dose–response relationships, interactions with cardiometabolic genetic risk, and metabolomic mediators[1]

by Xianglian Cai, MD, Yiwei Zhang, MD, Ziliang Ye, MD, Yanjun Zhang, MD, Sisi Yang, MD, Xiaoqin Gan, MD, Hao Xiang, MD, Yu Huang, MD, Yiting Wu, MD, Dan Chen, MD, Xiaolong Liang, MD, Xianhui Qin, MD,[2] *Yuanyuan Zhang, MD*

1. Supplementary material
2. Correspondence to: Xianhui Qin, MD, Division of Nephrology, Nanfang Hospital, Southern Medical University, Guangzhou 510515, China. [Email: pharmaqin@126.com]

Figure S1. Flow chart of study participants
Figure S2. Directed acyclic graph (DAG).
Figure S3. Distribution of lifetime night shifts' duration (A) and frequency (B)
Figure S4. Association of the lifetime night shifts' duration (A), frequency (B) with Incident Chronic Kidney Disease (CKD) Risk
Figure S5. Subgroup analyses of night shift work with risk of CKD
Figure S6. Subgroup analyses of lifetime night shifts' duration with risk of CKD
Figure S7. Subgroup analyses of lifetime night shifts' average frequency with risk of CKD
Figure S8. Association between polygenic risk score for T2DM (A), HT (B), and CVD (C) with incident CKD risk

Table S1. Baseline characteristics of participants between work questionnaire non-respondents and respondents.
Table S2. Combined effects of lifetime night shift work duration and frequency on incident chronic kidney disease
Table S3. Association between lifetime night shift work exposure (stratification by median) and incident CKD risk
Table S4. Time difference in CKD onset across current night shift work and lifetime night shifts
Table S5. Sensitivity analysis for the association of current night shift work and lifetime night shifts with incident chronic kidney disease
Table S6. Association of current night shift work and lifetime night shifts with incident CKD after further adjust other covariates and using Fine-Gray competing risk model
Table S7. Interaction of CKM genetic risk and night shift work on the risk of CKD
Table S8. Plasma metabolites selected by the LASSO regression to create metabolic signature score for night shift work
Table S9. Relationship and mediating role of night shift-related plasma metabolites with CKD risk and its linear regression coefficients for each plasma metabolitea

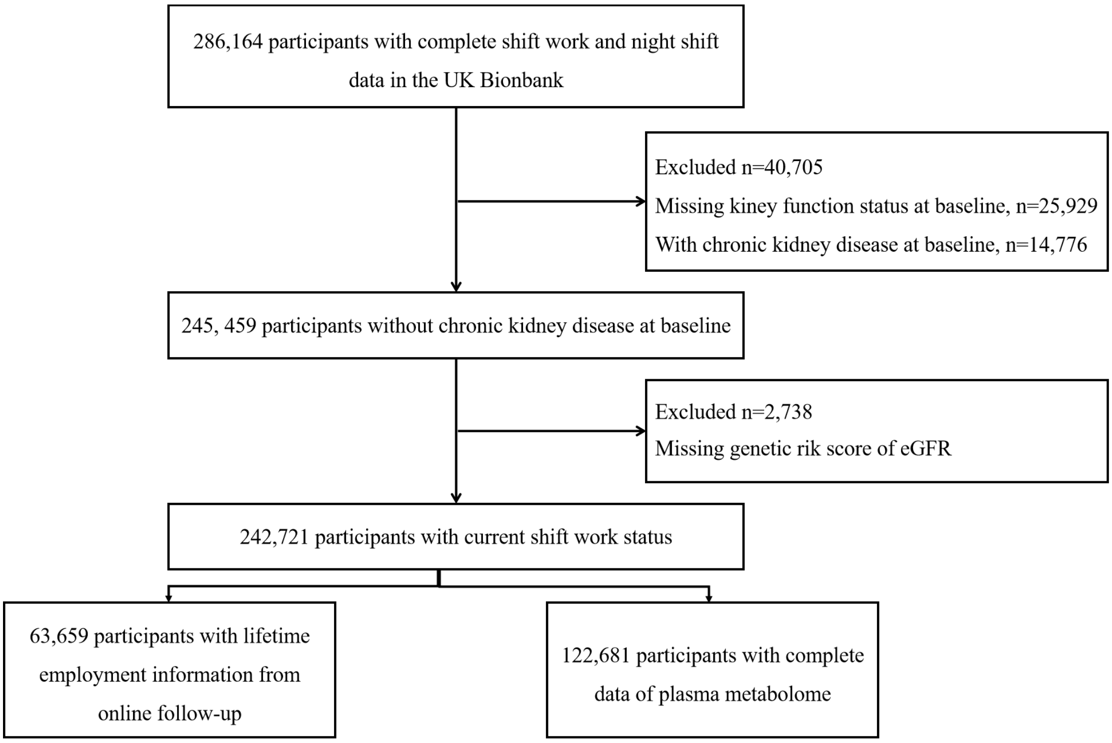

Figure S1. Flow chart of study participants

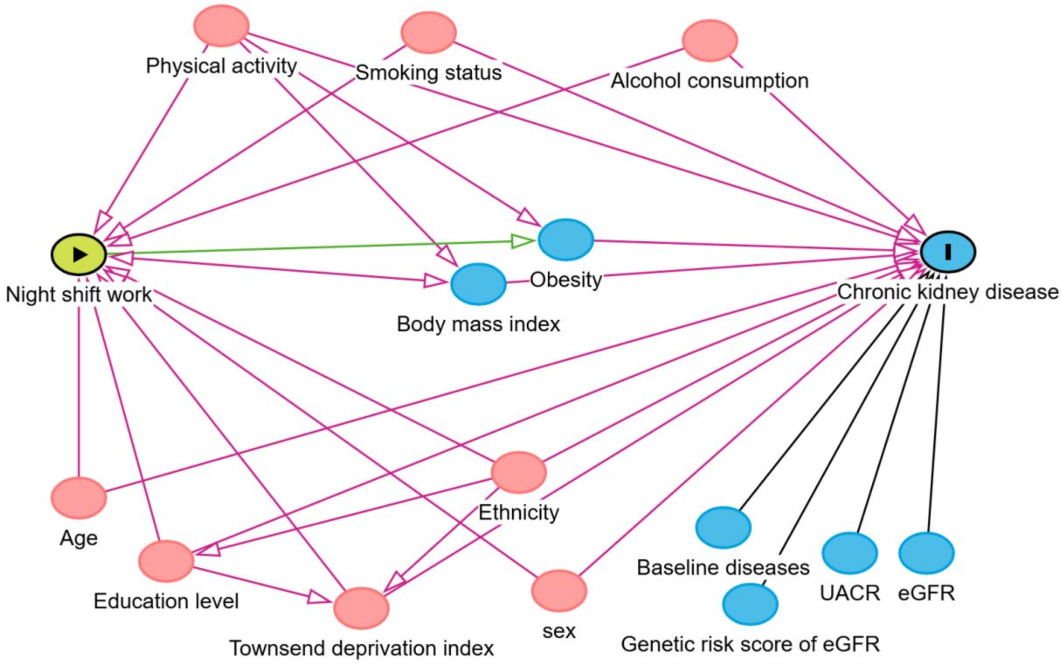

Figure S2. Directed acyclic graph (DAG).

Abbreviations: eGFR, estimated glomerular filtration rate; UACR, urine albumin-to-creatinine ratio.

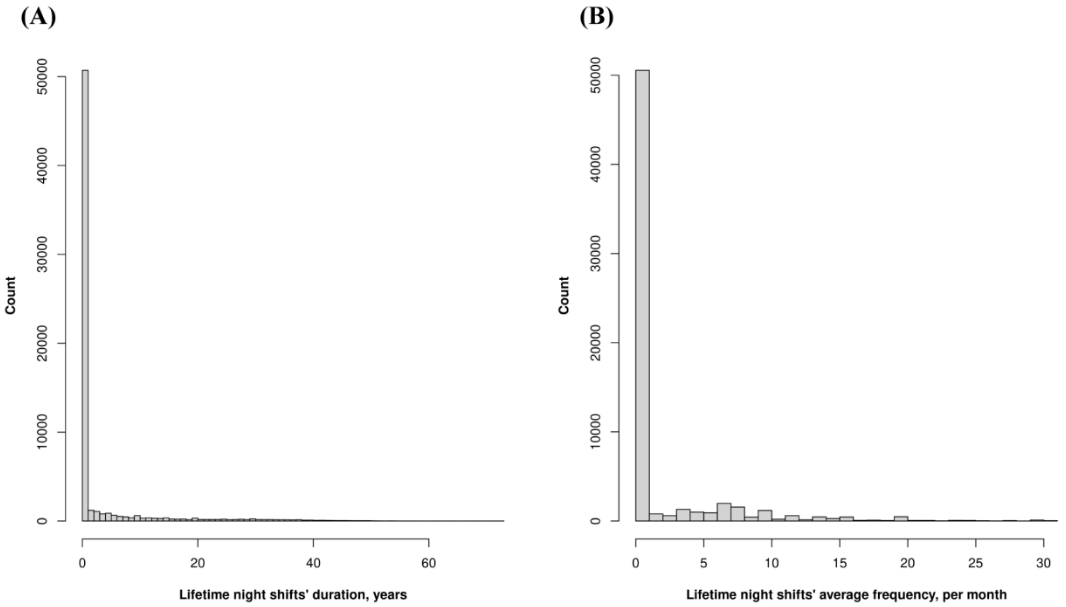

Figure S3. Distribution of lifetime night shifts' duration (A) and frequency (B)

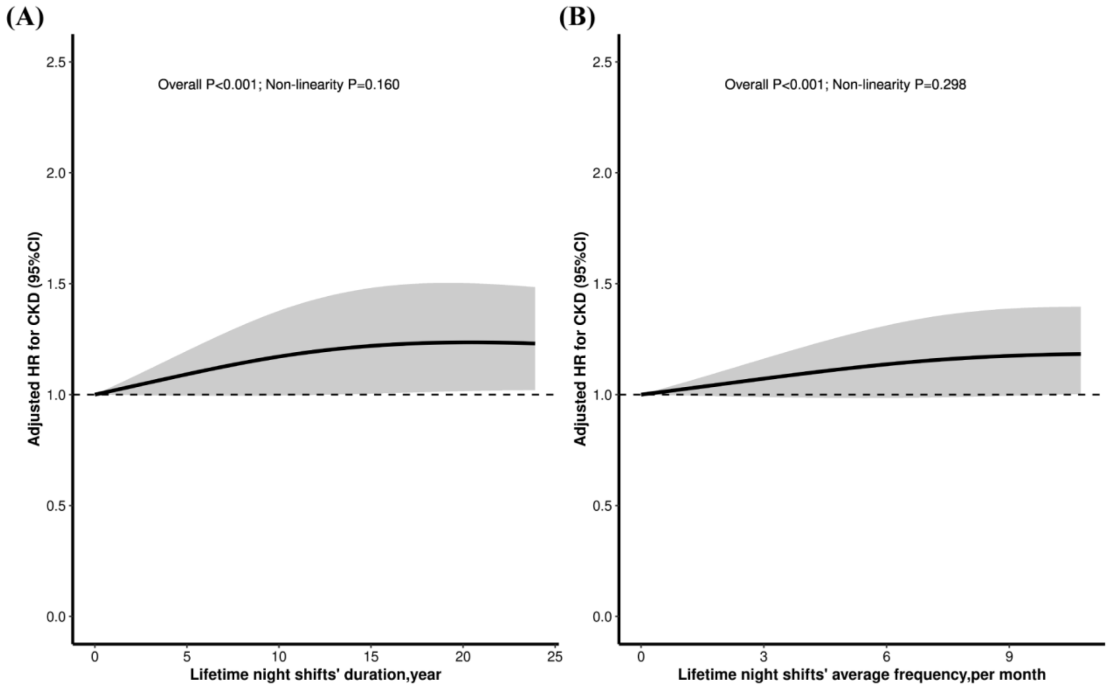

Figure S4. Association of the lifetime night shifts' duration (A), frequency (B) with Incident Chronic Kidney Disease (CKD) Risk*

*Adjusted for age, sex, ethnicity, Townsend deprivation index, education, body mass index, physical activity, smoking, alcohol, baseline diseases (hypertension, diabetes, high cholesterol), estimated glomerular filtration rate (eGFR), urine albumin-to-creatinine ratio, and genetic risk score of eGFR.

	Day workers (reference)	Shift with rare/some night	Shift with usually/always night	Day workers vs. Shift with rare/some night	Day workers vs. Shift with usually/always night	
	**Event (%)**	**Event (%)**	**Event (%)**	**HR (95%CI)**	**HR (95%CI)**	**P for interaction**
**N**	**4525 (2.3)**	**859 (2.7)**	**270 (2.9)**	**1.17 (1.08, 1.26)**	**1.25 (1.10, 1.41)**	
**Subgroup**						
**Age, years**						**0.687**
<65	3883 (2.0)	770 (2.5)	247 (2.7)	1.15 (1.07, 1.25)	1.20 (1.05, 1.37)	
≥65	642 (7.7)	89 (8.6)	23 (14.0)	1.04 (0.84, 1.30)	1.23 (0.81, 1.87)	
**Sex**						**0.162**
Female	2159 (2.0)	388 (2.6)	117 (3.4)	1.21 (1.09, 1.35)	1.42 (1.18, 1.71)	
Male	2366 (2.5)	471 (2.7)	153 (2.6)	1.13 (1.02, 1.25)	1.14 (0.96, 1.34)	
**Body mass index, kg/m^2**						**0.473**
<30	2896 (1.8)	497 (2.1)	147 (2.3)	1.21 (1.09, 1.33)	1.34 (1.13, 1.58)	
≥30	1629 (3.8)	362 (4.1)	123 (4.4)	1.14 (1.02, 1.28)	1.17 (0.97, 1.41)	
**Smoking status**						**0.159**
Ever	2221 (2.7)	474 (3.2)	148 (3.4)	1.24 (1.13, 1.38)	1.34 (1.13, 1.58)	
Never	2304 (2.0)	385 (2.2)	122 (2.5)	1.10 (0.98, 1.22)	1.17 (0.98, 1.41)	
**Alcohol consumption**						**0.052**
No	1492 (2.9)	337 (3.1)	143 (4.1)	1.10 (0.98, 1.24)	1.41 (1.18, 1.67)	
Yes	3033 (2.0)	522 (2.4)	127 (2.2)	1.22 (1.11, 1.34)	1.11 (0.93, 1.33)	
**Social isolation**						**0.227**
No	3822 (2.2)	695 (2.6)	213 (2.8)	1.19 (1.09, 1.29)	1.30 (1.13, 1.50)	
Yes	653 (2.3)	142 (2.6)	49 (2.9)	1.05 (0.88, 1.26)	1.05 (0.78, 1.40)	
**Chronotype**						**0.187**
Evening	1466 (2.1)	260 (2.3)	133 (3.2)	1.05 (0.92, 1.20)	1.34 (1.12, 1.60)	
Morning	2630 (2.3)	494 (2.8)	108 (2.8)	1.20 (1.09, 1.32)	1.21 (1.00, 1.47)	
**Hypertension**						**0.627**
No	1420 (1.3)	255 (1.5)	84 (1.8)	1.15 (1.01, 1.32)	1.36 (1.09, 1.70)	
Yes	3105 (3.4)	604 (3.9)	186 (4.0)	1.17 (1.07, 1.28)	1.20 (1.03, 1.39)	
**Diabetes**						**0.456**
No	3971 (2.0)	722 (2.3)	226 (2.6)	1.15 (1.06, 1.25)	1.28 (1.11, 1.46)	
Yes	554 (7.8)	137 (9.3)	44 (8.9)	1.26 (1.04, 1.52)	1.12 (0.82, 1.52)	
**High cholesterol**						**0.849**
No	3247 (1.8)	608 (2.1)	184 (2.3)	1.18 (1.08, 1.28)	1.27 (1.09, 1.48)	
Yes	1278 (5.8)	251 (6.4)	86 (7.4)	1.14 (1.00, 1.31)	1.19 (0.96, 1.49)	
**CKD genetic risk**						**0.610**
Low	1771 (1.8)	368 (2.2)	107 (2.2)	1.14 (1.03, 1.25)	1.28 (1.09, 1.50)	
High	2754 (2.7)	491 (3.1)	163 (3.7)	1.21 (1.08, 1.35)	1.20 (0.98, 1.46)	

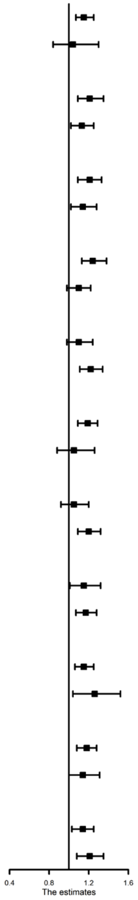

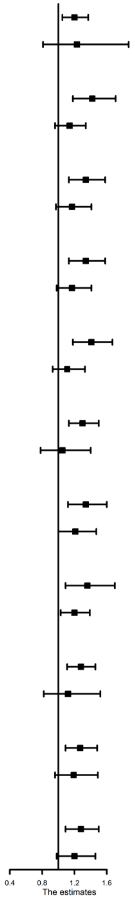

Figure S5. Subgroup analyses of night shift work with risk of CKD*

*Adjusted for age and sex, ethnicity, Townsend deprivation index, education, body mass index, physical activity, smoking, alcohol, baseline diseases (hypertension, diabetes, high cholesterol), estimated glomerular filtration rate, urine albumin-to-creatinine ratio, except when the variable was used as the subgroup.

Abbreviations: CI, confidence interval; CKD, chronic kidney disease; HR, hazard ratio.

	None (reference) Event (%)	<5 years Event (%)	≥5 years Event (%)	None vs. <5 years HR (95%CI)	None vs. ≥5 years HR (95%CI)	P for interaction
**N**	**902 (1.8)**	**74 (1.9)**	**226 (2.3)**	**0.98 (0.78, 1.25)**	**1.17 (1.01, 1.36)**	
**Subgroup**						
**Age, years**						**0.839**
<65	796 (1.7)	64 (1.7)	201 (2.1)	0.94 (0.73, 1.21)	1.12 (0.96, 1.32)	
≥65	106 (6.1)	10 (7.6)	25 (10.1)	1.14 (0.59, 2.18)	1.22 (0.78, 1.89)	
**Sex**						**0.919**
Female	464 (1.6)	32 (1.6)	78 (1.8)	1.04 (0.73, 1.49)	1.18 (0.93, 1.50)	
Male	438 (2.0)	42 (2.1)	148 (2.7)	0.94 (0.69, 1.30)	1.16 (0.96, 1.41)	
**Body mass index, kg/m^2**						**0.810**
<30	616 (1.5)	44 (1.4)	133 (1.8)	0.97 (0.72, 1.32)	1.22 (1.01, 1.47)	
≥30	286 (3.3)	30 (3.4)	93 (3.8)	1.06 (0.72, 1.54)	1.13 (0.89, 1.43)	
**Smoking status**						**0.593**
Ever	417 (2.2)	38 (2.1)	133 (3.0)	0.93 (0.67, 1.30)	1.24 (1.02, 1.52)	
Never	485 (1.6)	36 (1.7)	93 (1.7)	1.05 (0.74, 1.47)	1.10 (0.88, 1.37)	
**Alcohol consumption**						**0.385**
No	261 (2.2)	22 (2.1)	81 (3.0)	0.85 (0.55, 1.32)	1.31 (1.02, 1.68)	
Yes	641 (1.7)	52 (1.8)	145 (2.0)	1.05 (0.79, 1.39)	1.11 (0.93, 1.34)	
**Social isolation**						**0.978**
No	745 (1.8)	62 (1.9)	179 (2.2)	1.00 (0.77, 1.30)	1.17 (0.99, 1.38)	
Yes	147 (1.9)	12 (1.9)	45 (2.8)	0.95 (0.52, 1.71)	1.19 (0.85, 1.66)	
**Chronotype**						**0.684**
Evening	296 (1.8)	29 (2.0)	73 (2.0)	1.03 (0.70, 1.51)	1.06 (0.82, 1.37)	
Morning	520 (1.9)	39 (1.8)	131 (2.5)	0.97 (0.70, 1.35)	1.21 (0.99, 1.47)	
**Hypertension**						**0.757**
No	293 (1.0)	26 (1.1)	68 (1.3)	1.12 (0.75, 1.67)	1.18 (0.90, 1.54)	
Yes	609 (2.8)	48 (2.8)	158 (3.5)	0.92 (0.69, 1.24)	1.17 (0.97, 1.39)	
**Diabetes**						**0.120**
No	812 (1.7)	67 (1.7)	205 (2.2)	1.06 (0.82, 1.36)	1.21 (1.04, 1.42)	
Yes	90 (7.1)	7 (5.1)	21 (5.7)	0.57 (0.26, 1.22)	0.84 (0.52, 1.35)	
**High cholesterol**						**0.214**
No	679 (1.5)	56 (1.6)	152 (1.7)	1.10 (0.83, 1.44)	1.12 (0.94, 1.34)	
Yes	223 (4.8)	18 (4.1)	74 (6.4)	0.75 (0.46, 1.21)	1.29 (0.99, 1.68)	
**CKD genetic risk**						**0.768**
Low	340 (1.4)	30 (1.5)	93 (1.9)	0.98 (0.72, 1.33)	1.12 (0.92, 1.36)	
High	562 (2.2)	44 (2.2)	133 (2.7)	0.99 (0.68, 1.44)	1.25 (0.99, 1.58)	

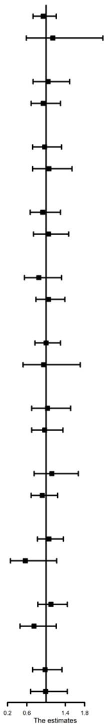

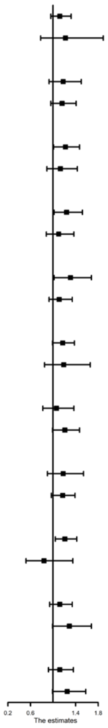

Figure S6. Subgroup analyses of lifetime night shifts' duration with risk of CKD*

*Adjusted for age and sex, ethnicity, Townsend deprivation index, education, body mass index, physical activity, smoking, alcohol, baseline diseases (hypertension, diabetes, high cholesterol), estimated glomerular filtration rate, urine albumin-to-creatinine ratio, except when the variable was used as the subgroup.

Abbreviations: CI, confidence interval; CKD, chronic kidney disease; HR, hazard ratio.

	None (reference) Event (%)	<3/month Event (%)	≥3/month Event (%)	None vs. <3/month HR (95%CI)	None vs. ≥3/month HR (95%CI)	P for interaction
**N**	**902 (1.8)**	**29 (1.7)**	**271 (2.2)**	**0.84 (0.58,1.22)**	**1.16 (1.01,1.33)**	
**Subgroup**						
**Age, years**						**0.640**
<65	796 (1.7)	24 (1.5)	241 (2.0)	0.79 (0.52, 1.18)	1.11 (0.96, 1.29)	
≥65	106 (6.1)	5 (10.0)	30 (9.1)	1.29 (0.52, 3.17)	1.18 (0.78, 1.77)	
**Sex**						**0.615**
Female	464 (1.6)	8 (1.0)	102 (1.8)	0.66 (0.33, 1.33)	1.20 (0.97, 1.49)	
Male	438 (2.0)	21 (2.3)	169 (2.6)	0.94 (0.60, 1.45)	1.13 (0.94, 1.35)	
**Body mass index, kg/m^2**						**0.853**
<30	616 (1.5)	18 (1.4)	159 (1.7)	0.80 (0.50, 1.29)	1.20 (1.01, 1.43)	
≥30	286 (3.3)	11 (3.1)	112 (3.7)	0.91 (0.50, 1.67)	1.13 (0.91, 1.41)	
**Smoking status**						**0.140**
Ever	417 (2.2)	21 (2.8)	150 (2.7)	1.12 (0.72, 1.74)	1.16 (0.96, 1.40)	
Never	485 (1.6)	8 (0.9)	121 (1.8)	0.51 (0.25, 1.02)	1.17 (0.95, 1.43)	
**Alcohol consumption**						**0.075**
No	261 (2.2)	16 (3.6)	87 (2.7)	1.36 (0.82, 2.26)	1.14 (0.89, 1.46)	
Yes	641 (1.7)	13 (1.0)	184 (2.1)	0.58 (0.33, 1.00)	1.17 (0.99, 1.38)	
**Social isolation**						**0.401**
No	745 (1.8)	21 (1.5)	220 (2.2)	0.75 (0.49, 1.16)	1.17 (1.01, 1.37)	
Yes	147 (1.9)	8 (2.8)	49 (2.5)	1.32 (0.64, 2.69)	1.10 (0.79, 1.52)	
**Chronotype**						**0.293**
Evening	296 (1.8)	12 (2.0)	90 (2.0)	0.96 (0.54, 1.71)	1.06 (0.84, 1.35)	
Morning	520 (1.9)	11 (1.2)	159 (2.4)	0.58 (0.32, 1.06)	1.22 (1.02, 1.47)	
**Hypertension**						**0.227**
No	293 (1.0)	5 (0.6)	89 (1.3)	0.50 (0.21, 1.20)	1.25 (0.99, 1.59)	
Yes	609 (2.8)	24 (3.1)	182 (3.4)	0.98 (0.65, 1.48)	1.12 (0.94, 1.32)	
**Diabetes**						**0.098**
No	812 (1.7)	25 (1.5)	247 (2.1)	0.84 (0.56, 1.25)	1.22 (1.05, 1.41)	
Yes	90 (7.1)	4 (6.7)	24 (5.4)	0.80 (0.29, 2.20)	0.74 (0.47, 1.16)	
**High cholesterol**						**0.431**
No	679 (1.5)	17 (1.1)	191 (1.8)	0.71 (0.44, 1.15)	1.17 (1.00, 1.38)	
Yes	223 (4.8)	12 (6.2)	80 (5.7)	1.15 (0.64, 2.06)	1.12 (0.87, 1.46)	
**CKD genetic risk**						**0.608**
Low	340 (1.4)	14 (1.7)	109 (1.8)	0.72 (0.43, 1.21)	1.13 (0.95, 1.35)	
High	562 (2.2)	15 (1.8)	162 (2.7)	1.04 (0.61, 1.77)	1.19 (0.96, 1.49)	

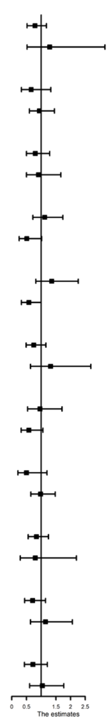

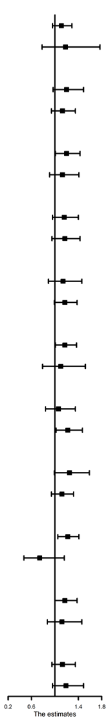

Figure S7. Subgroup analyses of lifetime night shifts' average frequency with risk of CKD*

*Adjusted for age and sex, ethnicity, Townsend deprivation index, education, body mass index, physical activity, smoking, alcohol, baseline diseases (hypertension, diabetes, high cholesterol), estimated glomerular filtration rate, urine albumin-to-creatinine ratio, except when the variable was used as the subgroup.

Abbreviations: CI, confidence interval; CKD, chronic kidney disease; HR, hazard ratio.

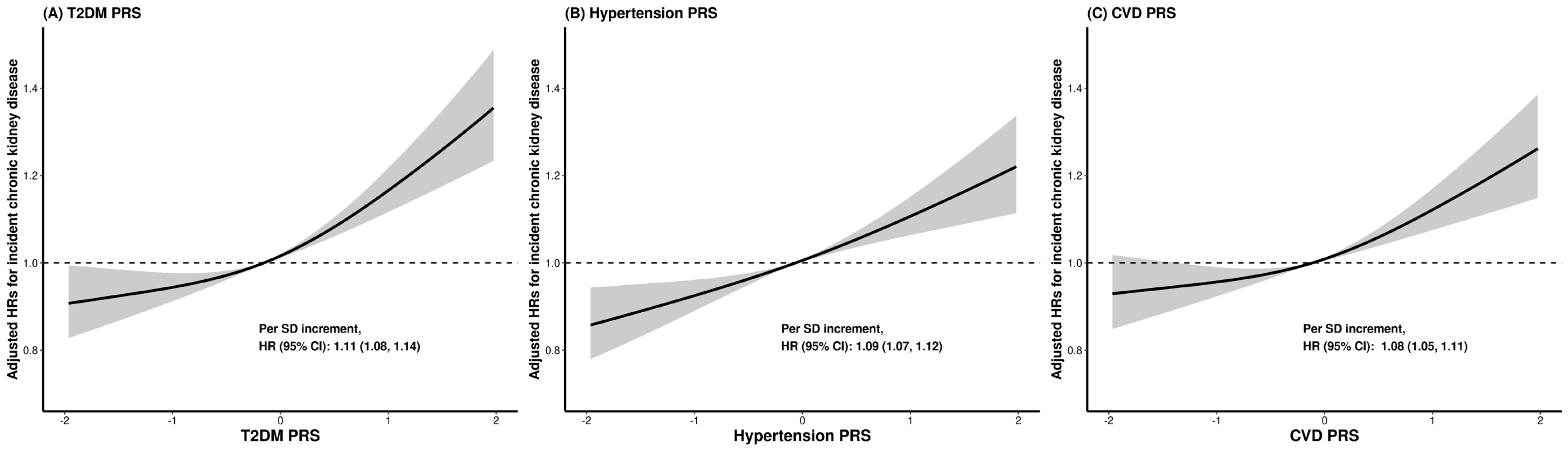

Figure S8. Association between polygenic risk score for T2DM (A), Hypertension (B), and CVD (C) with incident CKD risk*

*Adjusted for age and sex, ethnicity, Townsend deprivation index, education, body mass index, physical activity, smoking, alcohol, baseline diseases (hypertension, diabetes, high cholesterol), estimated glomerular filtration rate, urine albumin-to-creatinine ratio, except diabetes in (A), hypertension in (B, C).

Abbreviations: CI, confidence interval; CKD, chronic kidney disease; CVD, cardiovascular disease; ; HR, hazard ratio; PRS, polygenic risk score; T2DM, type 2 diabetes mellitus.

Table S1. Baseline characteristics of participants between work questionnaire non-respondents and respondents. *

	Overall	Work questionnaire non-respondents	Work questionnaire respondents
**N**	242,721	179,062	63,659
**Age, years**	52.64 (7.1)	52.56 (7.1)	52.87 (6.8)
**Sex, n (%)**			
Female	124,416 (51.3)	89,713 (50.1)	34,703 (54.5)
Male	118,305 (48.7)	89,349 (49.9)	28,956 (45.5)
**White, n (%)**	229,352 (94.5)	167,419 (93.5)	61,933 (97.3)
**Townsend deprivation index**	-1.37 (3.0)	-1.26 (3.1)	-1.70 (2.8)
**Body mass index, kg/m^2**	27.18 (4.6)	27.37 (4.7)	26.64 (4.5)
**Education, n (%)**			
College or university degree/vocational qualification	119,228 (49.4)	80,918 (45.5)	38,310 (60.3)
National examination at age 16	68,631 (28.4)	54,471 (30.6)	14,160 (22.3)
National examination at age 17-18	29,773 (12.3)	21,038 (11.8)	8,735 (13.7)
Others	23,659 (9.8)	21,302 (12.0)	2,357 (3.7)
**Moderate/vigorous physical activities > 4 days/week, n (%)**	130,704 (56.2)	98,135 (57.6)	32,569 (52.4)
**Smoking status, n (%)**			
Current	25,785 (10.7)	21,155 (11.8)	4,630 (7.3)
Never	139,243 (57.5)	100,807 (56.5)	38,436 (60.5)
Previous	77,071 (31.8)	56,573 (31.7)	20,498 (32.2)

**Alcohol, n (%)**			
<1/ week	66,727 (27.5)	51,286 (28.7)	15,441 (24.3)
1~2/ week	67,251 (27.7)	50,397 (28.2)	16,854 (26.5)
3~4/ week	60,582 (25.0)	43,013 (24.0)	17,569 (27.6)
>4/ week	48,022 (19.8)	34,237 (19.1)	13,785 (21.7)
**Sleep score**	3.08 (1.0)	3.06 (1.0)	3.16 (1.0)
**Social isolation, n (%)**	36,017 (15.0)	26,152 (14.8)	9,865 (15.6)
**History of disease, n (%)**			
Hypertension, n (%)	112,732 (46.6)	85,100 (47.7)	27,632 (43.5)
Diabetes, n (%)	9,073 (3.7)	7,301 (4.1)	1,772 (2.8)
High cholesterol, n (%)	27,277 (11.2)	21,061 (11.8)	6,216 (9.8)

*Variables are presented as Mean (standard deviation), or n (%).

Table S2. Combined effects of lifetime night shift work duration and frequency on incident chronic kidney disease *

	N	Case (%)	Model 1[a]		Model 2[b]	
			HR (95%CI)	*P* value	HR (95%CI)	*P* value
None	49831	902 (1.8)	Ref		Ref	
Low duration (<5 years) and low frequency (<3/month) of night shift	593	11 (1.9)	0.95 (0.52, 1.72)	0.863	0.92 (0.50, 1.66)	0.771
Low duration and high frequency of night shift	3383	63 (1.9)	1.06 (0.82, 1.37)	0.669	1.00 (0.77, 1.29)	0.984
High duration and low frequency of night shift	1090	18 (1.7)	0.87 (0.55, 1.39)	0.558	0.80 (0.50, 1.28)	0.357
High duration and high frequency of night shift	8762	208 (2.4)	1.35 (1.16, 1.57)	<0.001	1.22 (1.04, 1.42)	0.012

[a] **Model 1:** Adjusted for age and sex, ethnicity, Townsend deprivation index, education.

[b] **Model 2:** Additionally adjusted for body mass index, physical activity, smoking, alcohol, baseline diseases (hypertension, diabetes, high cholesterol), estimated glomerular filtration rate, urine albumin-to-creatinine ratio, genetic risk score of estimated glomerular filtration rate.

Abbreviations: CI, confidence interval; CKD, chronic kidney disease; HR, hazard ratio.

Table S3. Association between lifetime night shift work exposure (stratification by median) and incident CKD risk

	N	Case (%)	Model 1[a]		Model 2[b]	
			HR (95%CI)	*P* value	HR (95%CI)	*P* value
**Lifetime night shifts' duration**						
**Per-SD**	63659	1202 (1.9)	1.08 (1.03, 1.13)	0.003	1.04 (0.99, 1.09)	0.103
**Log-transformed**	63659	1202 (1.9)	1.09 (1.04, 1.15)	<0.001	1.05 (1.00, 1.11)	0.052
**Categories**						
None	49831	902 (1.8)	Ref		Ref	
<10 years	6809	134 (2.0)	1.13 (0.94, 1.35)	0.202	1.06 (0.88, 1.27)	0.547
≥10 years	7019	166 (2.4)	1.31 (1.11, 1.55)	0.002	1.17 (0.99, 1.39)	0.069
P for trend			<0.001		0.054	
**Lifetime night shifts' average frequency**						
**Per-SD**	63659	1202 (1.9)	1.08 (1.03, 1.13)	0.003	1.04 (0.99, 1.10)	0.084
**Log-transformed**	63659	1202 (1.9)	1.11 (1.04, 1.17)	<0.001	1.06 (1.00, 1.13)	0.053
**Categories**						
None	49831	902 (1.8)	Ref		Ref	
<7/month	5692	100 (1.8)	1.03 (0.84, 1.27)	0.748	0.99 (0.81, 1.23)	0.961
≥7/month	8136	200 (2.5)	1.34 (1.15, 1.57)	<0.001	1.19 (1.02, 1.39)	0.029
P for trend			<0.001		0.043	

[a] **Model 1:** Adjusted for age and sex, ethnicity, Townsend deprivation index, education.

[b] **Model 2:** Additionally adjusted for body mass index, physical activity, smoking, alcohol, baseline diseases (hypertension, diabetes, high cholesterol), estimated glomerular filtration rate, urine albumin-to-creatinine ratio, genetic risk score of estimated glomerular filtration rate.

Abbreviations: CI, confidence interval; CKD, chronic kidney disease; HR, hazard ratio.

Table S4. Time difference in CKD onset across current night shift work and lifetime night shifts

	N	Case (%)	Time to Earlier Incident (years)[a]
**Current night shift work**			
Day workers	201001	4525 (2.3)	Ref
Shift with rare/some night	32406	859 (2.7)	1.93 (1.18, 2.64)
Shift with usually/always night	9314	270 (2.9)	2.06 (0.75, 3.25)
**Lifetime night shifts' duration**			
None	49831	902 (1.8)	Ref
≥5 years	9852	226 (2.3)	1.24 (0.18, 2.21)
**Lifetime night shifts' average frequency**			
None	49831	902 (1.8)	Ref
≥3/month	12145	271 (2.2)	1.10 (0.12, 2.03)

[a] Adjusted for age and sex, ethnicity, Townsend deprivation index, education, body mass index, physical activity, smoking, alcohol, baseline diseases (hypertension, diabetes, high cholesterol), estimated glomerular filtration rate, urine albumin-to-creatinine ratio, genetic risk score of estimated glomerular filtration rate.

Abbreviations: CI, confidence interval; CKD, chronic kidney disease; HR, hazard ratio.

Table S5. Sensitivity analysis for the association of current night shift work and lifetime night shifts with incident chronic kidney disease.

	N	Event (%)	Adjusted HR (95%CI)*	*P* value
**Sensitivity analysis 1: Excluding participants who had an incident CKD diagnosed within 2 years after baseline**				
**Current night shift work**				
Day workers	200,638	4,162 (2.1)	Ref	
Shift with rare/some night	32,314	767 (2.4)	1.14 (1.05,1.23)	0.002
Shift with usually/always night	9,302	258 (2.8)	1.30 (1.14,1.48)	<0.001
P for trend			<0.001	
**Lifetime night shifts' duration**				
None	49,760	831 (1.7)	Ref	
<5 years	3,966	64 (1.6)	0.92 (0.71, 1.18)	0.510
≥5 years	9,840	214 (2.2)	1.20 (1.03, 1.40)	0.019
P for trend			0.035	
**Lifetime night shifts' average frequency**				
None	49,760	831 (1.7)	Ref	
<3/month	1,683	29 (1.7)	0.91 (0.63, 1.32)	0.611
≥3/month	12,123	271 (2.1)	1.15 (1.00, 1.33)	0.055
P for trend			0.067	
**Sensitivity analysis 2: Excluding participants with missing covariate data.**				
**Current night shift work**				
Day workers	190,842	4,208 (2.2)	Ref	
Shift with rare/some night	29,862	774 (2.6)	1.17 (1.08,1.26)	<0.001
Shift with usually/always night	8,404	232 (2.8)	1.22 (1.07,1.40)	0.003
P for trend			<0.001	
**Lifetime night shifts' duration**				
None	48,312	865 (1.8)	Ref	
<5 years	3,850	69 (1.8)	0.95 (0.74, 1.21)	0.654
≥5 years	9,483	214 (2.3)	1.16 (1.00, 1.35)	0.058
P for trend			0.085	
**Lifetime night shifts' average frequency**				
None	48,312	865 (1.8)	Ref	
<3/month	1,617	28 (1.7)	0.85 (0.58, 1.24)	0.389
≥3/month	11,716	255 (2.2)	1.14 (0.98, 1.31)	0.082
P for trend			0.106	
**Sensitivity analysis 3: Without adjusted for baseline diseases.**				

**Current night shift work**				
Day workers	201,001	4525 (2.3)	Ref	
Shift with rare/some night	32,406	859 (2.7)	1.16 (1.08, 1.25)	<0.001
Shift with usually/always night	9,314	270 (2.9)	1.27 (1.12, 1.44)	<0.001
P for trend			<0.001	
**Lifetime night shifts' duration**				
None	49,831	902 (1.8)	Ref	
<5 years	3,976	74 (1.9)	1.00 (0.79, 1.27)	0.982
≥5 years	9,852	226 (2.3)	1.17 (1.00, 1.36)	0.043
P for trend			0.053	
**Lifetime night shifts' average frequency**				
None	49,831	902 (1.8)	Ref	
<3/month	1,683	29 (1.7)	0.85 (0.59, 1.24)	0.407
≥3/month	12,145	271 (2.2)	1.16 (1.01, 1.33)	0.037
P for trend			0.05	
**Sensitivity analysis 4: Excluding participants still currently employed.**				
**Lifetime night shifts' duration**				
None	22,009	560 (2.5)	Ref	
<5 years	1,562	44 (2.8)	0.99(0.73,1.35)	0.974
≥5 years	3,904	134 (3.4)	1.19(0.98,1.44)	0.084
P for trend			0.101	
**Lifetime night shifts' average frequency**				
None	22,009	560 (2.5)	Ref	
<3/month	700	20 (2.9)	0.96(0.61,1.50)	0.845
≥3/month	4,766	158 (3.3)	1.16(0.97,1.39)	0.112
P for trend			0.124	

*Additionally adjusted for body mass index, physical activity, smoking, alcohol, baseline diseases (hypertension, diabetes, high cholesterol), estimated glomerular filtration rate, urine albumin-to-creatinine ratio, genetic risk score of estimated glomerular filtration rate.

Abbreviations: CI, confidence interval; CKD, chronic kidney disease; HR, hazard ratio.

Table S6. Association of current night shift work and lifetime night shifts with incident CKD after further adjust other covariates and using Fine-Gray competing risk model.

	N	Event (%)	Analysis 1	Analysis 2	Analysis 3	Analysis 4	Analysis 5
**Current night shift work**							
Day workers	201,001	4,525 (2.3)	Ref	Ref	Ref	Ref	Ref
Shift with rare/some night	32,406	859 (2.7)	1.14 (1.06,1.23)	1.16 (1.08,1.25)	1.17 (1.08,1.26)	1.17 (1.09, 1.26)	1.16 (1.08, 1.25)
Shift with usually/always night	9,314	270 (2.9)	1.20 (1.06,1.36)	1.23 (1.08,1.39)	1.25 (1.10,1.42)	1.26 (1.11, 1.43)	1.25 (1.10, 1.42)
P for trend			<0.001	<0.001	<0.001	<0.001	<0.001
**Lifetime night shifts' duration**							
None	49,831	902 (1.8)	Ref	Ref	Ref	Ref	Ref
<5 years	3,976	74 (1.9)	0.98 (0.77,1.24)	0.98 (0.77,1.25)	0.99 (0.78,1.26)	0.98 (0.78, 1.25)	0.98 (0.77, 1.26)
≥5 years	9,852	226 (2.3)	1.15 (0.99,1.34)	1.17 (1.01,1.36)	1.17 (1.01,1.36)	1.17 (1.01, 1.36)	1.17 (1.01, 1.36)
P for trend			0.094	0.056	0.051	0.0646	0.054
**Lifetime night shifts' average frequency**							
None	49,831	902 (1.8)	Ref	Ref	Ref	Ref	Ref
<3/month	1,683	29 (1.7)	0.83 (0.57,1.20)	0.84 (0.58,1.22)	0.85 (0.59,1.24)	0.84 (0.58, 1.22)	0.84 (0.59, 1.21)
≥3/month	12,145	271 (2.2)	1.14 (0.99,1.31)	1.16 (1.01,1.33)	1.16 (1.01,1.33)	1.16 (1.01, 1.33)	1.16 (1.01, 1.33)
P for trend			0.089	0.057	0.051	0.052	0.058

Sensitivity analysis 1: Further adjusted for work type.

Sensitivity analysis 2: Further adjusted for sleep score.

Sensitivity analysis 3: Further adjusted for social isolation.

Sensitivity analysis 4: Further adjusted for weekly working hours.

Sensitivity analysis 5: Used the Fine-Gray competing risk model to account for the competing risk of death.

Abbreviations: CI, confidence interval; CKD, chronic kidney disease; HR, hazard ratio.

Table S7. Interaction of CKM genetic risk and night shift work on the risk of CKD

	N	Event (%)	HR (95%CI)[a]	*P* value	*P* for interaction
**Type 2 diabetes mellitus**					0.866
**Low T2DM genetic risk**					
Day workers	101311	2013 (2.0)	Ref		
Shift with rare/some night	15453	350 (2.3)	1.15 (1.03,1.29)	0.016	
Shift with usually/always night	4384	103 (2.3)	1.19 (0.98,1.46)	0.080	
**High T2DM genetic risk**					
Day workers	99378	2504 (2.5)	Ref		
Shift with rare/some night	16871	505 (3.0)	1.17 (1.06,1.29)	0.002	
Shift with usually/always night	4899	164 (3.3)	1.28 (1.09,1.50)	0.003	
**Hypertension**					0.646
**Low Hypertension genetic risk**					
Day workers	100884	2042 (2.0)	Ref		
Shift with rare/some night	15791	385 (2.4)	1.19 (1.07,1.33)	0.002	
Shift with usually/always night	4473	121 (2.7)	1.31 (1.09,1.57)	0.005	
**High Hypertension genetic risk**					
Day workers	99805	2475 (2.5)	Ref		
Shift with rare/some night	16533	470 (2.8)	1.14 (1.03,1.26)	0.009	
Shift with usually/always night	4810	146 (3.0)	1.18 (1.00,1.40)	0.054	
**Cardiovascular disease**					0.380
**Low CVD genetic risk**					
Day workers	100942	2051 (2.0)	Ref		
Shift with rare/some night	15727	398 (2.5)	1.23 (1.10,1.37)	<0.001	
Shift with usually/always night	4479	114 (2.5)	1.20 (1.00,1.46)	0.055	
**High CVD genetic risk**					
Day workers	99747	2466 (2.5)	Ref		

Shift with rare/some night	16597	457 (2.8)	1.11 (1.01,1.23)	0.039
Shift with usually/always night	4804	153 (3.2)	1.26 (1.07,1.49)	0.006

[a]Adjusted for age and sex, ethnicity, Townsend deprivation index, education, body mass index, physical activity, smoking, alcohol, baseline diseases (hypertension, diabetes, high cholesterol), estimated glomerular filtration rate, urine albumin-to-creatinine ratio, genetic risk score of estimated glomerular filtration rate, except diabetes in (T2DM), hypertension in (Hypertension, CVD).

Abbreviations: CI, confidence interval; CKD, chronic kidney disease; CKM, Cardiovascular-Kidney-Metabolic; HR, hazard ratio.

Table S8. Plasma metabolites selected by the LASSO regression to create metabolic signature score for night shift work

Metabolites	Group	Coefficients
Free Cholesterol to Total Lipids in Very Large HDL percentage	Very large HDL ratios	0.0193
Docosahexaenoic Acid to Total Fatty Acids percentage	Fatty acids	-0.0130
Omega-3 Fatty Acids to Total Fatty Acids percentage	Fatty acids	-0.0085
Average Diameter for HDL Particles	Lipoprotein particle sizes	-0.0054
Albumin	Fluid balance	-0.0038
Cholesteryl Esters in Very Large HDL	Very large HDL	-0.0036
Phospholipids to Total Lipids in Large HDL percentage	Large HDL ratios	0.0032
Glycoprotein Acetyls	Inflammation	0.0030
Free Cholesterol to Total Lipids in Medium HDL percentage	Medium HDL ratios	-0.0020

Abbreviations: HDL, high density lipoproteins.

Table S9. Relationship and mediating role of night shift-related plasma metabolites with CKD risk and its linear regression coefficients for each plasma metabolite[a]

Metabolites	Group	*β* (95%CIs)	HRs (95% CIs)	Proportions mediated (%) (95% CIs)
Docosahexaenoic Acid to Total Fatty Acids percentage	Fatty acids	-0.0151 (-0.0181, -0.0122)	0.88 (0.85,0.91)	5.33 (3.07, 12.00)
Omega-3 Fatty Acids to Total Fatty Acids percentage	Fatty acids	-0.0137 (-0.0166, -0.0109)	0.88 (0.85,0.92)	4.94 (2.75, 11.00)
Albumin	Fluid balance	-0.0075 (-0.0103, -0.0047)	0.90 (0.87,0.94)	2.36 (1.12, 5.00)
Glycoprotein Acetyls	Inflammation	0.0070 (0.0040,0.0099)	1.10 (1.05,1.14)	1.70 (0.73, 4.00)
Cholesteryl Esters in Very Large HDL	Very large HDL	-0.0142 (-0.0175, -0.0109)	0.99 (0.95,1.04)	_
Free Cholesterol to Total Lipids in Very Large HDL percentage	Very large HDL ratios	0.0155 (0.0122,0.0188)	1.01 (0.98,1.05)	_
Phospholipids to Total Lipids in Large HDL percentage	Large HDL ratios	0.0140 (0.0110,0.0170)	1.03 (0.99,1.07)	_
Free Cholesterol to Total Lipids in Medium HDL percentage	Medium HDL ratios	-0.0098 (-0.0131, -0.0066)	0.98 (0.94,1.02)	_
Average Diameter for HDL Particles	Lipoprotein particle sizes	-0.0132 (-0.0166, -0.0098)	0.99 (0.95,1.04)	_

[a]Adjusted for age and sex, ethnicity, Townsend deprivation index, education, body mass index, physical activity, smoking, alcohol, baseline diseases (hypertension, diabetes, high cholesterol), estimated glomerular filtration rate, urine albumin-to-creatinine ratio, genetic risk score of estimated glomerular filtration rate.

Abbreviations: CI, confidence interval; CKD, chronic kidney disease; HR, hazard ratio; HDL, high density lipoproteins.
